# Emerging roles of circular RNAs in stem cells

**DOI:** 10.1016/j.gendis.2022.05.015

**Published:** 2022-05-31

**Authors:** Mengru Wang, Juan Wu, Pan Wu, Yuhong Li

**Affiliations:** Department of Cell Biology, Army Medical University, Chongqing 400038, China

**Keywords:** Circular RNA, Differentiation, Proliferation, Stem cell, Stem cell-derived exosomes

## Abstract

Circular RNAs (circRNAs) are a novel class of noncoding RNAs that widely exist in eukaryotes. As a new focus in the field of molecular regulation, circRNAs have attracted much attention in recent years. Previous studies have confirmed that circRNAs are associated with many physiological and pathological processes. CircRNAs also participate in the regulation of stem cells. Stem cells have the properties of self-renewal and differentiation, which make stem cell therapy popular. CircRNAs may serve as new targets in stem cell therapy due to their regulation in stem cells. However, the underlying relationships between circRNAs and stem cells are still being explored. In this review, we briefly summarize the effects of circRNAs on stem cells, in the context of biological activities, aging and apoptosis, and aberrant changes. Moreover, we also examine the biological roles of stem cell-derived exosomal circRNAs. We believe our review will provide insights into the effects of circRNAs on stem cells.

## Introduction

Circular RNAs (circRNAs), which have a covalently closed-loop structure, are a novel class of RNAs that are abundant in eukaryocytes. CircRNAs are circular products of precursor mRNA (pre-mRNA) formed by back-splicing, in which a downstream 5′ donor site is joined to an upstream 3′ acceptor site.^1^ This process mainly leads to the formation of exonic circRNAs or uncommonly forms exon–intron circRNAs.^2^ The appearance of intronic circRNAs benefits from the lariat intermediate structure.^3^ In addition, transcription termination failure and chromosomal translocations also contribute to the biogenesis of circRNAs.^4^^,^^5^ CircRNA biogenesis is coupled with pre-mRNA transcription by Pol II and competes with its linear counterparts. Biogenesis can also be regulated by core spliceosomal machinery, cis elements, RNA-binding proteins (RBPs), and circRNA turnover.^6^ Although circRNAs were first discovered in 1976,^7^ their underlying role was not truly understood until 2013, when circRNAs were found to play a role by sponging miRNAs.^8^^,^^9^ To date, researchers revealed that circRNAs could be translated into proteins, regulate transcription and interact with proteins besides sponging miRNAs.^10^ Due to advances in RNA sequencing technology, we found that circRNAs were closely related to many physiological and pathological conditions.^6^ CircRNAs also play essential roles in the regulation of disease processes such as cancer, cardiovascular disease, and renal disease,^11^, ^12^, ^13^ and these unprecedented discoveries may change current therapeutic strategies in the future.

It is well known that stem cells have self-renewal and multidirectional differentiation potential, play critical roles in growth and development in humans. Furthermore, stem cell dysfunction is highly associated with many diseases. For example, hematopoietic stem cell (HSC) dysfunction can directly lead to aplastic anemia (AA). Thus, an increasing number of people are turning their attention to stem cell therapy. In cell therapy, stem cells can be used to replace damaged cells or regenerate organs, and in drug development, disease-specific cell lines can also be used.^14^ At present, HSC transplantation (HSCT) has been widely used to treat a variety of hematological diseases.^15^ Stem cell therapy has brought new hope for treating certain diseases.

However, to utilize the potential abilities of stem cells for therapy, we must determine the mechanisms associated with their growth and development. CircRNAs, which are a new focus in the field of molecular regulation, have attracted much attention for their effects on stem cells. In recent years, many researchers have reported the important roles of circRNAs in regulating stem cell fate.^16^ CircRNAs usually act as miRNA sponges to regulate stem cell differentiation.^17^ For example, circFOXP1 could promote osteogenic differentiation in adipose-derived mesenchymal stem cells (ADSCs) by sponging miR-33a-5p.^18^ In addition, circRNAs can regulate transcription and translation,^10^ but these functions have not been clear in stem cells until now. CircRNAs can affect stem cells in many ways, including in the context of biological activities, aging and apoptosis, and aberrant changes. Moreover, stem cell-derived exosomes that contain circRNAs can also have vital effects on organs or tissues. Human umbilical cord mesenchymal stem cell-derived exosomes (hucMSC-Exos) prevent pyroptosis and repair ischemic muscle injury by releasing circHIPK3, which can enhance the expression of FOXO3a.^19^ These results suggests that stem cell therapy can target circRNAs. However, the distinct relationships between circRNAs and stem cells are not clear.

**Figure 1:**
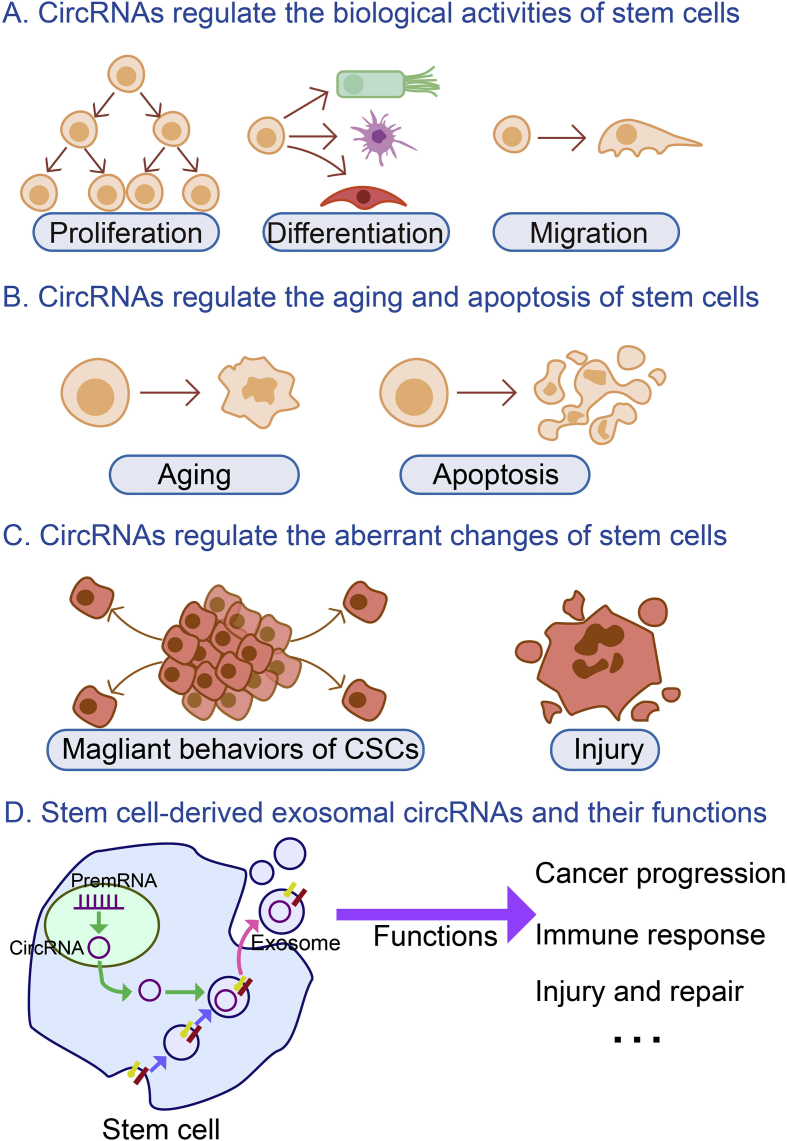
Effects of circRNAs on stem cells. Abbreviations: circRNA, circular RNA; CSC, cancer stem cell; premRNA, precursor mRNA.

In this review, we introduce the changes induced by circRNAs in stem cells (Fig. 1), including effects on biological activities, aging and apoptosis, aberrant changes and the effects of stem cell-derived exosomal circRNAs.

## CircRNAs regulate the biological activities of stem cells

### The effects of circRNAs on stem cell proliferation

The high proliferation capacity of stem cells is critical to the maintenance of tissue and organ homeostasis. The proliferation of stem cells is necessary for tissue or organ repair and self-renewal. In recent years, many researchers have found that circRNAs can promote stem cell proliferation. In human periodontal ligament stem cells (PDLSCs), the circRNA CDR1as was shown to promote proliferation by sponging miR-7 to activate the ERK signaling pathway in a lipopolysaccharide-induced inflammatory microenvironment.^20^ In addition, circMAP3K11 could also facilitate the proliferation of PDLSCs in an inflammatory microenvironment by sponging miR-511.^21^ The functions of CDR1as and circMAP3K11 in PDLSCs may promote periodontal tissue regeneration and reduce the injury caused by periodontitis. In mesenchymal stem cells (MSCs), many studies have verified the important role of circRNAs in determining their fate.^22^ In fact, MSCs perform good therapeutic effects on many diseases like bone and cartilage, neurological, cardiovascular, and autoimmune diseases.^23^ In regard to promoting the proliferation of MSCs, hsa_circ_0066523 promotes the proliferation of bone marrow-derived mesenchymal stem cells (BMSCs) by repressing phosphatase and tensin homolog (PTEN), thereby activating the AKT pathway.^24^ Mmu_circRNA_003795 can promote BMSC proliferation through calcitonin gene-related peptide (CGRP) by increasing FOS like 2 AP1 transcription factor subunit (FOSL2) expression by sponging miR-504-3p.^25^ CircRNA_0001052 accelerates BMSC proliferation by sponging miR-124-3p through the Wnt4/beta-catenin pathway in low-level laser irradiation treatment (LLLI).^26^ For clinical application, LLLI is a potential method to stimulate BMSC proliferation in patients. Circ_FBLN1, circ-DAB1 and circPVT1 also play positive roles in BMSC proliferation and have essential effects on BMSC differentiation or apoptosis at the same time.^27^, ^28^, ^29^ CDR1as was confirmed to take a part in maintaining proliferation and differentiation of hucMSCs.^30^ In myoblasts, circSVIL could facilitate bovine myoblast proliferation by inhibiting STAT1 phosphorylation.^31^ In another study, circSVIL functioned as a miR-203 sponge and upregulated the mRNA levels of c-JUN and MEF2C to promote the proliferation of myoblasts in chickens.^32^ CircHIPK3 could act as a sponge of miR-7 to promote murine myoblast proliferation and function as a sponge of miR-30a-3p to promote chicken myoblast proliferation.^33^^,^^34^ Circ-ZNF609 was also found to have a positive effect on myoblast proliferation; surprisingly, researchers found that circ-ZNF609 could be translated into protein because it contains an open reading frame.^35^ In addition, circHUWE1, circTTN, circINSR, circRILPL1, circCPE and circFGFR2 can also promote myoblast proliferation by sponging corresponding microRNAs.^36^, ^37^, ^38^, ^39^, ^40^, ^41^ In MC3T3-E1 cells, the circRNA AFF4 acts as a sponge of miR-7223-5p, thereby promoting MC3T3-E1 cell proliferation and inhibiting apoptosis.^42^

CircRNAs can also inhibit the cell proliferation. For instance, circ-Smad5 can restrain the proliferation of JB6 cells by reducing the activation of wnt/lef/cyclind1 signaling.^43^ Regarding stem cell proliferation, circTTC3 inhibits the proliferation of neural stem cells (NSCs) through the miR-372-3p/Toll-like receptor 4 (TLR4) axis, and the depletion of circTTC3 promotes proliferation and upregulates nestin and β-tubulin III expression in NSCs. Moreover, the depletion of circTTC3 reduces cerebral infarction, neurological score, and brain water content in mouse model, which implies that circTTC3 can be a target in treating cerebral ischemia/reperfusion injury during stroke.^44^ CircRNA_25487 inhibits BMSC proliferation by sequestering miR-134-3p, which can inhibit bone repair in trauma-induced osteonecrosis of the femoral head.^45^ In myoblasts, circMYL1, circSNX29 and circFUT10 can reduce myoblast proliferation by sponging miR-2400, miR-744 and miR-133a, respectively.^46^, ^47^, ^48^ The detailed studies were summarized in Table 1.

**Table 1:** Roles of circRNAs in stem cell proliferation.

CircRNAs	Species	Cell types	Mechanisms	Target genes or pathways	Effects on proliferation	Ref
CDR1as	Homo sapiens	PDLSC	Sponge miR-7	ERK signaling pathway	Promote	^20^
CircMAP3K11	Homo sapiens	PDLSC	Sponge miR-511	TLR4	Promote	^21^
Hsa_circ_0066523	Homo sapiens	BMSC	Repress PTEN	AKT signaling pathway	Promote	^24^
Mmu_circRNA_003795	Mus	BMSC	Sponge miR-504-3p	FOSL2	Promote	^25^
CircRNA_0001052	Mus	BMSC	Sponge miR-124-3p	Wnt4/beta-catenin pathway	Promote	^26^
Circ_FBLN1	Homo sapiens	BMSC	Sponge let-7i-5p	FZD4, Wnt/beta-catenin pathway	Promote	^27^
Circ-DAB1	Homo sapiens	BMSC	Sponge miR-1270 and miR-944	NOTCH/RBPJ pathway	Promote	^28^
CircPVT1	Rattus	BMSC	Sponge miR-21-5p	Smad7/TGFbeta pathway	Promote	^29^
CDR1as	Homo sapiens	hucMSC	Unknown	STFs	Promote	^30^
CircSVIL	Bos taurus	Myoblast	Inhibit STAT1 phosphorylation	Downstream signal cascade of STAT1	Promote	^31^
	Gallus gallus		Sponge miR-203	MEF2C and c-JUN	Promote	^32^
CircHIPK3	Mus	Myoblast	Sponge miR-7	TCF12	Promote	^33^
	Gallus gallus		Sponge miR-30a-3p	Unknown	Promote	^34^
Circ-ZNF609	Mus and Homo sapiens	Myoblast	Unknown	Unknown	Promote	^35^
CircHUWE1	Bos taurus	Myoblast	Sponge miR-29b	AKT signaling pathway	Promote	^36^
CircTTN	Bos taurus	Myoblast	Sponge miR-432	IGF2/PI3K/AKT pathway	Promote	^37^
CircINSR	Bos taurus	Myoblast	Sponge miR-34a	Bcl-2 and CyclinE2	Promote	^38^
CircRILPL1	Bos taurus	Myoblast	Sponge miR-145	IGF1R/PI3K/AKT pathway	Promote	^39^
CircCPE	Bos taurus	Myoblast	Sponge miR-138	FOXC1	Promote	^40^
CircFGFR2	Gallus gallus	Myoblast	Sponge miR-133a-5p and miR-29b-1-5p	Unknown	Promote	^41^
CircRNA AFF4	Mus	MC3T3-E1 cell	Sponge miR-7223-5p	PIK3R1	Promote	^42^
Circ-Smad5	Mus	JB6 cell	Unknown	Wnt/lef/cyclind1 signaling	Inhibit	^43^
CircTTC3	Mus	NSC	Sponge miR-372-3p	TLR4	Inhibit	^44^
CircRNA_25487	Homo sapiens	BMSC	Sponge miR-134-3p	p21	Inhibit	^45^
CircMYL1	Bos taurus	Myoblast	Sponge miR-2400	Unknown	Inhibit	^46^
CircSNX29	Bos taurus	Myoblast	Sponge miR-744	Wnt5a/Ca (2+) signaling pathway	Inhibit	^47^
CircFUT10	Bos taurus	Myoblast	Sponge miR-133a	Unknown	Inhibit	^48^
CircFAM188B	Gallus gallus	SMSC	Encode circFAM188B-103aa	Unknown	Promote	^94^
CircFNDC3AL	Gallus gallus	SMSC	Sponge miR-204	BCL9	Promote	^95^
CircPPP1R13B	Gallus gallus	SMSC	Sponge miR-9-5p	IGF/PI3K/AKT signaling pathway	Promote	^96^
CircITSN2	Gallus gallus	Myoblast	Sponge miR-218-5p	LMO7	Promote	^100^
CircEch1	Bos taurus	Myoblast	Unknown	Unknown	Inhibit	^101^
CircLMO7	Bos taurus	Myoblast	Sponge miR-378a-3p	Unknown	Promote	^102^

BMSC, bone marrow-derived mesenchymal stem cell; c-JUN, c-Jun N-terminal kinase; FOSL2, FOS like 2 AP-1 transcription factor subunit; FZD4, frizzled class receptor 4; hucMSC, mesenchymal stem cell derived from human umbilical cord; IGF1R, insulin-like growth factor 1 receptor; IGF2, insulin-like growth factor 2; MC3T3-E1 cell, mouse embryo osteoblast precursor cell; MEF2C, myocyte enhancer factor 2 C; NSC, neural stem cell; PDLSC, periodontal ligament stem cell; PI3K, phosphatidylinositol 3-kinase; PIK3R1, phosphoinositide-3-kinase regulatory subunit 1; PTEN, phosphatase and tensin homolog; RBPJ, recombination signal-binding protein for immunoglobulin kappa J region; SMSC, skeletal muscle satellite cell; STAT1, signal transducing activator of transcription 1; STFs, stemness transcription factors; TCF12, transcription factor 12; TGF, transforming growth factor; TLR4, toll-like receptor 4.

### The effects of circRNAs on stem cell differentiation

**Figure 2:**
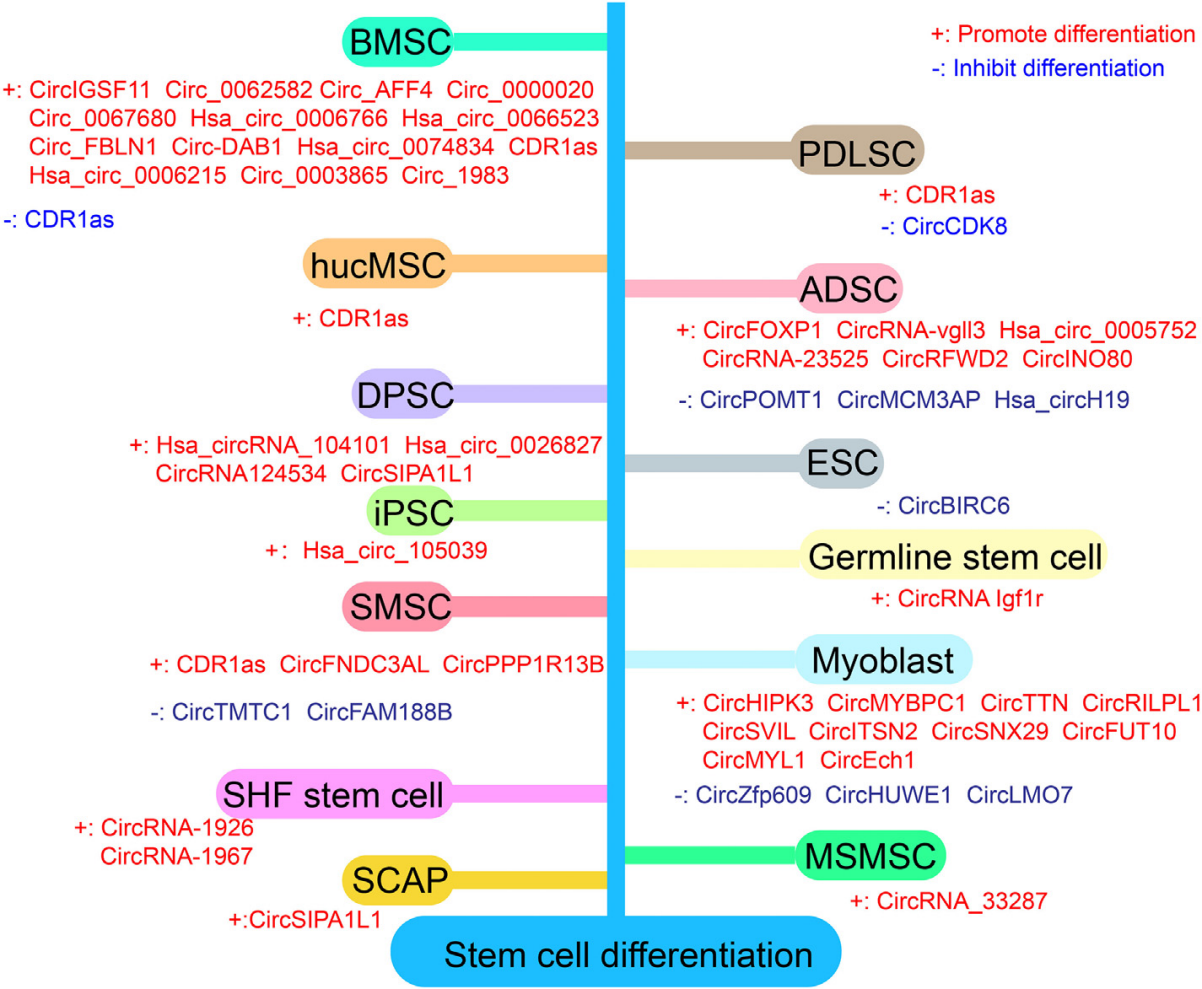
CircRNAs in stem cell differentiation. The figure shows that the role of circRNAs in the differentiation of different stem cells. CircRNAs that can promote differentiation were marked in red and circRNAs that can inhibit differentiation were marked in blue. CDR1as can promote adipogenic differentiation while suppresses osteogenic differentiation in BMSCs. Abbreviations: ADSC, adipose-derived mesenchymal stem cell; BMSC, bone marrow-derived mesenchymal stem cell; DPSC, dental pulp stem cell; ESC, embryonic stem cell; HSC, hematopoietic stem cell; hucMSC, human umbilical cord mesenchymal stem cell; iPSC, induced pluripotent stem cell; MSMSC, maxillary sinus membrane stem cell; PDLSC, periodontal ligament stem cell; SCAP, stem cells from apical papilla; SHF, secondary hair follicle; SMSC, skeletal muscle satellite cell.

It is universally acknowledged that stem cell differentiation is indispensable for human development. Stem cell-directed differentiation enables the specific functions of organs and tissues. Mechanistically, cell differentiation involves the selective expression of genes in time and space. At the molecular level, the role of circRNAs in stem cell differentiation has been a research hotspot for several years. Here, we summarize previous research on the role of circRNAs in the differentiation of various types of stem cells (Fig. 2).

### The effects of circRNAs on mesenchymal stem cell (MSC) differentiation

MSCs are pluripotent stem cells that have the potential to differentiate into a variety of tissue cells and have strong immunomodulatory effects. In stem cell therapy, MSCs are one of the most widely used and safest stem cells at present. Moreover, MSCs can be derived from various tissues, such as bone marrow, adipose tissue, umbilical cord and placental chorionic plates. Here, we introduce the function of circRNAs in the differentiation of MSCs derived from different tissues.

In BMSCs, circRNAs modulate osteogenic differentiation. The dysfunction of BMSC osteogenic differentiation is associated with a range of diseases including osteoporosis, osteoarthritis and osteonecrosis. Microarray analysis revealed changes in circRNAs in BMSCs during osteoblast differentiation and showed that 3938 circRNAs were upregulated and 1505 were downregulated in BMSCs at day 7 (D7) compared with day 0 (D0). The study validated the expression of the hub miRNA, miR-199b-5p, which was associated with circIGSF11. Silencing circIGSF11 facilitated osteoblast differentiation and increased the expression of miR-199b-5p.^49^ In fact, circRNAs typically act as sponges for miRNAs and reduce their effects on stem cell differentiation.^17^ In addition, circRNAs often have specific expression patterns. Circ_0062582 overexpression significantly promotes osteogenic differentiation by upregulating core-binding factor subunit beta (CBFB) expression by targeting miR-145.^50^ CBFB plays an important role in stabilizing RUNX family proteins, which are essential for osteogenic differentiation.^51^ By sponging miR-135a-5p, circ_AFF4 can trigger high expression of FNDC5/Irisin, which activates the SMAD1/5 pathway to promote osteogenic differentiation.^52^ Circ_0000020 promotes osteogenic differentiation by sponging miR-142-5p, which upregulates the bone morphogenetic protein BMP2.^53^ Similarly, circ_0067680 and hsa_circ_0006766 also accelerate osteogenic differentiation in BMSCs through the circ_0067680/miR-4429/CTNNB1/Wnt/β-catenin pathway and hsa_circ_0006766/miR-4739/Notch2 regulatory axis, respectively.^54^^,^^55^ In addition to promoting osteogenic differentiation, some circRNAs also have other effects on BMSCs. For example, hsa_circ_0066523 facilitates osteogenic differentiation and proliferation in BMSCs by suppressing PTEN expression.^24^ Circ_FBLN1 and circ-DAB1 can also promote proliferation and osteogenic differentiation through their specific expression patterns.^27^^,^^28^ In BMSCs from patients with nonunion, hsa_circ_0074834 is found to be downregulated by high-throughput assay. As a ceRNA for miR-942-5p, hsa_circ_0074834 not only accelerates osteogenic differentiation in BMSCs but also promotes the osteogenesis-angiogenesis coupling process by increasing ZEB1 and VEGF expression.^56^ Analogously, hsa_circ_0006215, which decreases in BMSCs from patients with osteoporosis, expedites osteogenic differentiation in BMSCs and strengthens osteogenesis–angiogenesis coupling by competitively binding to miR-942-5p and regulating RUNX2 and VEGF expression. Hsa_circ_0006215 also promote bone regeneration in a cortical bone defect model.^57^ In addition, changes in the extracellular environment of BMSCs can also cause changes in circRNAs, which may affect differentiation. Melatonin can suppress circ_0003865 expression and increase miR-3653-3p expression, and miR-3653-3p inhibits the expression of growth arrest-specific gene 1 (GAS1) to promote osteogenic differentiation. Moreover, circ_0003865 silencing repressed osteoporosis development in mouse model.^58^ In dicalcium silicate microparticle (C2S)-treated BMSCs, circ_1983 expression was elevated, and circ_1983 can sponge miR-6931 to facilitate osteogenic differentiation by increasing GAS7 and RUNX2 expression.^59^ Surface mechanical attrition treatment (SMAT) with titanium can also alter osteogenic differentiation by altering the expression of circRNAs. Bioinformatics analysis using miRWalk3.0 software showed that 51 circRNAs were predicted to construct ceRNA networks with osteogenesis-related mRNAs and miRNAs.^60^ It is worth noting that circRNAs can affect the adipogenic differentiation of BMSCs as well. CircRNA CDR1as promotes adipogenesis and suppresses osteogenesis in BMSCs in steroid-induced osteonecrosis of the femoral head through the CDR1as/miR-7-5p/WNT5B axis.^61^ The axis provides a novel target for treating osteonecrosis.

In ADSCs, circRNAs also influence osteogenic differentiation. A circRNA microarray showed that a total of 171 circRNAs were upregulated and 119 were downregulated during induced osteogenic differentiation in ADSCs compared with noninduced cells.^62^ This result hints at the importance of circRNAs in the osteogenic differentiation of ADSCs. CircFOXP1 acts as a miR-33a-5p sponge to upregulate the expression of FOXP1, which controls several differentiation pathways, thereby promoting osteogenic differentiation in ADSCs.^18^ CircRNA-vgll3 facilitates ADSC osteogenic differentiation by activating the integrin α5 (Itga5) pathway by sequestering miR-326-5p. In a rat bone defects model, circRNA-vgll3-modified ADSCs obviously enhance new bone formation.^63^ This confirms the value of circRNA-vgll3 in healing injured bone. Hsa_circ_0005752 can sponge miR-496 to mediate ADSC osteogenic differentiation, and RUNX3 can enhance the level of hsa_circ_0005752.^64^ CircRNA-23525 regulates RUNX2 expression by targeting miR-30a-3p and promotes osteogenic differentiation in ADSCs.^65^ Similarly, circRFWD2 and circINO80 can interact with hsa-miR-6817-5p in ADSCs and play a positive role in recombinant NELL-1-induced osteogenesis.^66^ Conversely, circPOMT1 and circMCM3AP inhibit the osteogenic differentiation of ADSCs by targeting miR-6881-3p.^67^ In the context of ADSC adipogenic differentiation, knockdown of hsa_circH19 promotes this process because hsa_circH19 restrains the expression of polypyrimidine tract-binding protein 1 (PTBP1). In fact, hsa_circH19 is upregulated in patients with metabolic syndrome and correlates with lipid-related parameters.^68^ The suppression of adipogenic differentiation of ADSC is one of the main causes of disorders associated with metabolic syndrome.^69^ Targeting hsa_circH19 may improve symptoms of metabolic syndrome.

In hucMSCs, CDR1as may play an important role during adipogenesis and osteogenesis. After CDR1as knockdown, the expression of stemness transcription factors (STFs) was downregulated, and the adipogenesis and osteogenesis potential of hucMSCs was impaired.^30^ During the differentiation of hucMSCs into cardiomyocyte-like cells induced by 5-aza, RT-PCR showed that a total of 226 circRNAs were differentially expressed, and gene ontology (GO) analysis identified a number of cell proliferation- and differentiation-related physiological processes and pathways.^70^

### The effects of circRNAs on PDLSC differentiation

PDLSCs are an important focus in periodontal regeneration and alveolar bone repair. During PDLSC osteogenic differentiation, an analysis revealed that 147 lncRNAs and 1382 circRNAs were predicted to bind with 148 common miRNAs and compete for miRNA binding sites with 744 messenger RNAs, and these miRNAs had been predicted to regulate the pluripotency of stem cells.^71^ Another analysis showed that the expression of circRNAs during PDLSC osteogenic differentiation was stage-specific. The expression profiles of circRNAs are altered during different stages of osteogenic differentiation.^72^ Thus, the roles of circRNAs are worthy of intensive study. CDR1as was markedly upregulated, while miR-7 was significantly downregulated during PDLSC osteoblastic differentiation.^73^ The study also showed that the 3′ untranslated region (UTR) of growth and differentiation factor-5 (GDF5) contained miR-7 binding sites. GDF5 is closely associated with osteogenic differentiation.^74^ Therefore, CDR1as acts upstream of miR-7 and inhibits the effect of miR-7 by targeting GDF5, which enhances Smad1/5/8 and p38 MAPK phosphorylation and promotes osteoblastic differentiation.^73^ In addition, by sponging miR-7, CDR1as can increase the expression of the stemness-related gene KLF4 to promote PDLSC multidifferentiation and migration.^75^ In cobalt chloride (CoCl2) induced hypoxia, circCDK8 can induce autophagy and apoptosis through mTOR signaling and then suppress the osteogenic differentiation in PDLSCs.^76^ Hypoxia of periodontal tissues is usually an outcome of periodontitis. Therefore, circCDK8 could be a therapeutic target of periodontitis accompanied by hypoxia.

### The effects of circRNAs on dental pulp stem cell (DPSC) differentiation

DPSCs are fibroblasts isolated from dental pulp tissue and are a kind of MSC. DPSCs are not an ideal candidate stem cells for prosthodontics but also play important roles in the repair of other tissues due to their excellent capacity for multidifferentiation. During the odontogenic differentiation of hDPSCs, a study identified 43 upregulated and 144 downregulated circRNAs by using qRT-PCR and microarrays. Among these, hsa_circRNA_104101 was shown to promote the odontogenic differentiation of hDPSCs.^77^ In DPSC osteogenic differentiation, hsa_circ_0026827 can act a positive role by sponging miR-188-3p, which can decrease the Beclin-1-mediated autophagy and increase the expression of RUNX1.^78^ CircRNA124534 can also promote DPSC osteogenic differentiation by activating the β-catenin pathway by binding to miR-496.^79^ Moreover, circSIPA1L1 promotes osteogenesis in DPSCs by sponging miR-617 and further targeting Smad3, which is beneficial for osteogenic differentiation.^80^ Hsa_circ_0026827, circRNA124534, and circSIPA1L1 could be a candidate target in DPSCs for treating diseases associated with bone formation.

### The effects of circRNAs on embryonic stem cell (ESC) differentiation

ESCs are highly undifferentiated cells. ESCs can theoretically differentiate into all tissue and organ types. An analysis of hESC differentiation showed that the dominant forms of the lncRNAs RMST and FIRRE are circular, suggesting that the roles of circRNAs in hESCs cannot be neglected.^81^ CircBIRC6 can suppress hESC differentiation by directly interacting with miR-34a and miR-145 because miR-34a/miR-145 modulate target genes that maintain pluripotency.^82^ In addition, the RBP FUS participates in circRNA expression in murine ESCs and plays a role in ESC-derived motor neuron differentiation. In this study, Lorenzo Errichelli et al used RNA sequencing analysis and showed that 3894 circRNAs among 2097 known genes were expressed in ESC-derived motor neurons.^83^

### The effects of circRNAs on hematopoietic stem cell (HSC) differentiation

HSCs can differentiate into a variety of myeloid cells and lymphocytes and have strong self-renewal ability. HSCT has become an important therapeutic method and has been used to treat many serious diseases. However, the role of circRNAs in HSCs remains unclear. Xia et al showed that the circRNA cia-cGAS can maintain homeostasis in the context of cGAS-mediated HSC exhaustion. Cia-cGAS has a strong affinity for cGAS and inhibits cGAS-mediated production of type I IFNs in long-term HSCs.^84^ An analysis of circRNA expression in HSCs revealed that circRNAs were widely expressed. As hematopoiesis continues, the expression of circRNAs is altered. Moreover, circRNA expression is cell type specific during hematopoietic differentiation. High expression levels of circRNAs are observed in red blood cells (RBCs) and platelets. Intriguingly, circRNA expression is reduced in aging RBCs.^85^ CircRNA may play an important role in the function of hemocyte. However, the underlying mechanism and function remain to be explored.

### The effects of circRNAs on induced pluripotent stem cell (iPSC) differentiation

iPSCs have a strong capacity to differentiate into cells of all three germ layers, similar to ESCs, but iPSCs are easily obtained from somatic cells. Thus, the therapeutic application of iPSCs has become a hotspot but has faced a number of challenges.^86^ In regard to the effects of circRNAs on iPSCs, a circRNA map of iPSCs of fetal origin showed the differences between human iPSCs from cord blood multipotent stromal cells and human iPSCs from adult skin fibroblasts. The study showed that the 9 out of 10 top circRNAs were predicted to interact with 27 miRNAs in hiPSCs from cord blood multipotent stromal cells, but their functions are still unknown.^87^ In hiPSC-derived cardiomyocytes, a study showed more enriched expression of circRNAs than in undifferentiated hiPSCs. Moreover, circSLC8A1, circCACNA1D, circSPHKAP and circALPK2 may be biomarkers of cardiomyocytes due to their specificity.^88^ CircSLC8A1 abnormally upregulated in heart tissues from patients with dilated cardiomyopathy. Furthermore, in hiPSC-derived cardiomyocytes, hsa_circ_105039 can sponge miR-17 and upregulate cyclinD2 expression to promote this process.^89^

### The effects of circRNAs on germline stem cell differentiation

High-throughput sequencing and RT-PCR revealed 18573 novel lncRNAs and 18822 circRNAs in murine germline stem cells. In addition, 8115 mRNAs, 3996 lncRNAs and 921 circRNAs exhibited sex-biased expression, which may be associated with sex-specific properties. The study also showed that lncRNA Meg3 and circRNA Igf1r can act as sponges of miRNA-15a-5p and increase the expression of the target genes Inha, Acsl3, Kif21b, and Igfbp2.^90^ These genes may participate in many biological processes. CircGFRalpha1 is an N6-methyladenosine (m6A)-modified circRNA that is stage-specifically expressed during female germline stem cells development. CircGFRalpha1 sponges miR-449 to enhance GFRalpha1 expression and activate the GDNF signaling pathway, thus facilitating female germline stem cell self-renewal. Moreover, the m6A writer METTL14 can promote the cytoplasmic export of m6A-modified circGFRalpha1.^91^

### The effects of circRNAs on skeletal muscle satellite cell (SMSC) differentiation

SMSCs are the precursor cells of myoblasts and are a specific kind of adult stem cell in skeletal muscle. SMSCs are usually in a static state and are only activated when skeletal muscle is injured. CDR1as, which has attracted much attention, also plays a role in SMSC differentiation. CDR1as can bind to miR-7 to increase the expression of insulin-like growth factor-1 receptor (IGF1R) and promote SMSC differentiation in goat fetuses.^92^ CircTMTC1 can inhibit chicken SMSC differentiation by sponging miR-128-3p.^93^ CircFAM188B can repress SMSC differentiation and induce SMSC proliferation. Surprisingly, circFAM188B encodes a protein called circFAM188B-103aa because circFAM188B contains an open reading frame. The function of circFAM188B-103aa is consistent with that of circFAM188B.^94^ CircFNDC3AL and circPPP1R13B can facilitate SMSC proliferation and differentiation by targeting miR-204 and miR-9-5p, respectively.^95^^,^^96^ Therefore, circRNA has potential clinical application value in promoting myogenesis and repair of muscle injury.

### The effects of circRNAs on myoblast differentiation

Myoblasts are essential for skeletal muscle development and regeneration. Myoblasts are easy to identify, culture and reproduce, and the studies of circRNAs in myoblasts are notably. It has been mentioned previously that circRNAs can impact myoblast proliferation; in fact, circRNAs also affect myoblast differentiation. CircRNAs can sponge miRNAs in myoblast differentiation as well. Circ-HIPK3 sponges miR-124 and miR-379 to promote the differentiation of C2C12 myoblasts.^97^ CircMYBPC1 can directly bind to miR-23a to enhance the expression of myosin heavy chain (MyHC) and promote bovine myoblast differentiation.^98^ In contrast, by sponging miR-194-5p, circZfp609 can increase the expression of BCL2 associated transcription factor 1 (BCLAF1) to inhibit C2C12 myoblast differentiation.^99^ However, in many times, circRNAs can simultaneously regulate myoblast proliferation and differentiation. One case is that circRNAs synchronously facilitate myoblast proliferation and differentiation. These circRNAs include circTTN, circRILPL1, circSVIL, circHIPK3, circFGFR2, and circITSN2, which all play positive roles by sponging miRNAs.^32^, ^33^, ^34^^,^^37^^,^^39^^,^^41^^,^^100^ CircRNAs can also asynchronously regulate myoblast proliferation and differentiation. For example, circSNX29, circFUT10, circMYL1 and circEch1 can induce myoblast differentiation while reducing myoblast proliferation.^46^, ^47^, ^48^^,^^101^ In contrast, circHUWE1 and circLMO7 can facilitate myoblast proliferation and inhibit myoblast differentiation at the same time.^36^^,^^102^

### The effects of circRNAs on the differentiation of other stem cells

In maxillary sinus membrane stem cells (MSMSCs), circRNA_33287 can facilitate osteogenic differentiation and increase ectopic bone formation *in vivo*. The underlying mechanism is that circRNA_33287 can sponge miR-214-3p and increase the expression of RUNX3.^103^ In secondary hair follicle (SHF) stem cells, circRNAs act as molecular sponges for miRNAs to regulate differentiation. This can provide new method for the treatment of hair loss. During the differentiation of goat SHF stem cells into the hair follicle lineage, circRNA-1926 can promote cyclin dependent kinase 19 (CDK19) expression by sponging miR-14a/b-3p.^104^ CircRNA-1967 also participates by sponging miR-93-3p to enhance lymphoid enhancer binding factor 1 (LEF1) expression.^105^ Many circRNAs are upregulated during the differentiation of epidermal stem cells (EpSCs) to keratinocytes, as shown by RNA-seq analysis. Compared to host genes, upregulated circRNAs have more binding sites for argonaute 2 (AGO2), and these circRNAs may function as miRNA sponges. One study also showed that circZNF91 contains 24 target sites for miR-23b-3p, which is essential for keratinocyte differentiation. Moreover, upregulated circRNAs are unlikely to be regulated by DNA methylation.^106^ During the osteogenic differentiation of stem cells from apical papilla (SCAPs), 333 upregulated circRNAs and 317 downregulated circRNAs were identified by RNA-sequencing. In addition, 10 circRNAs, 21 miRNAs and mRNAs may construct competing endogenous RNA networks.^107^ By targeting miR-204-5p, circSIPA1L1 upregulates ALPL expression and promotes osteogenic differentiation.^108^ NSCs can differentiate into neurons, astrocytes and oligodentrites, promoting neural regeneration and repairing damage in the brain.^109^ An analysis showed that 12 circRNAs might play regulatory roles during NSC differentiation and suggested the underlying mechanisms of circRNA-mRNA in this process.^110^ Hsa_circ_0002468 can promote neural differentiation in human neuroblastoma differentiation model SH-SY5Y cells by sponging miR-561, and miR-561 can facilitate the expression of E2F transcription factor 8 (E2F8).^111^ The specific roles of circRNAs in stem cell differentiation were showed in Table 2.

**Table 2:** Roles of circRNAs in stem cell differentiation.

CircRNAs	Species	Cell types	Mechanisms	Target genes or pathways	Effects on differentiation	Ref
CircIGSF11	Homo sapiens	BMSC	Sponge miR-199b-5p	Unknown	Promote osteogenic differentiation	^49^
Circ_0062582	Homo sapiens	BMSC	Sponge miR-145	CBFB	Promote osteogenic differentiation	^50^
Circ_AFF4	Homo sapiens	BMSC	Sponge miR-135a-5p	SMAD1/5 pathway	Promote osteogenic differentiation	^52^
Circ_0000020	Rattus	BMSC	Sponge miR-142-5p	BMP2	Promote osteogenic differentiation	^53^
Circ_0067680	Homo sapiens	BMSC	Sponge miR-4429	CTNNB1/Wnt/beta-catenin pathway	Promote osteogenic differentiation	^54^
Hsa_circ_0006766	Homo sapiens	BMSC	Sponge miR-4739	Notch2	Promote osteogenic differentiation	^55^
Hsa_circ_0066523	Homo sapiens	BMSC	Repress PTEN	AKT signaling pathway	Promote osteogenic differentiation	^24^
Circ_FBLN1	Homo sapiens	BMSC	Sponge let-7i-5p	FZD4, Wnt/beta-catenin pathway	Promote osteogenic differentiation	^27^
Circ-DAB1	Homo sapiens	BMSC	Sponge miR-1270 and miR-944	NOTCH/RBPJ pathway	Promote osteogenic differentiation	^28^
Hsa_circ_0074834	Homo sapiens	BMSC	Sponge miR-942-5p	ZEB1, VEGF	Promote osteogenic differentiation and osteogenesis–angiogenesis coupling	^56^
Hsa_circ_0006215	Homo sapiens	BMSC	Sponge miR-942-5p	RUNX2, VEGF	Promote osteogenic differentiation and osteogenesis–angiogenesis coupling	^57^
Circ_0003865	Homo sapiens	BMSC	Sponge miR-3653-3p	GAS1	Promote osteogenic differentiation	^58^
Circ_1983	Mus	BMSC	Sponge miR-6931	GAS7 and RUNX2	Promote osteogenic differentiation	^59^
CDR1as	Homo sapiens	BMSC	Sponge miR-7-5p	WNT5B	Promote adipogenic differentiation Inhibit osteogenic differentiation	^61^
CircFOXP1	Homo sapiens	ADSC	Sponge miR-33a-5p	FOXP1	Promote osteogenic differentiation	^18^
CircRNA-vgll3	Rattus	ADSC	Sponge miR-326-5p	Itga5 pathway	Promote osteogenic differentiation	^63^
Hsa_circ_0005752	Homo sapiens	ADSC	Sponge miR-496	MDM2	Promote osteogenic differentiation	^64^
CircRNA-23525	Mus	ADSC	Sponge miR-30a-3p	RUNX2	Promote osteogenic differentiation	^65^
CircRFWD2 CircINO80	Homo sapiens	ADSC	Sponge hsa-miR-6817-5p	Unknown	Promote osteogenic differentiation	^66^
CircPOMT1 CircMCM3AP	Homo sapiens	ADSC	Sponge miR-6881-3p	BMPs signaling pathway	Inhibit osteogenic differentiation	^67^
Hsa_circH19	Homo sapiens	ADSC	Bind to PTBP1	Unknown	Inhibit adipogenic differentiation	^68^
CDR1as	Homo sapiens	hucMSC	Unknown	STFs	Promote osteogenic differentiation and adipogenic differentiation	^30^
CDR1as	Homo sapiens	PDLSC	Sponge miR-7	GDF5	Promote osteogenic differentiation	^73^
CDR1as	Homo sapiens	PDLSC	Sponge miR-7	KLF4	Promote multidifferentiation	^75^
CircCDK8	Homo sapiens	PDLSC	Unknown	mTOR signaling, autophagy	Inhibit osteogenic differentiation	^76^
Hsa_circRNA_104101	Homo sapiens	DPSC	Unknown	Unknown	Promote odontogenic differentiation	^77^
Hsa_circ_0026827	Homo sapiens	DPSC	Sponge miR-188-3p	Beclin1, RUNX1	Promote osteogenic differentiation	^78^
CircRNA124534	Homo sapiens	DPSC	Sponge miR-496	β-catenin pathway	Promote osteogenic differentiation	^79^
CircSIPA1L1	Homo sapiens	DPSC	Sponge miR-617	Smad3	Promote osteogenic differentiation	^80^
CircBIRC6	Homo sapiens	ESC	Sponge miR-34a and miR-145	Pluripotency-associated genes	Inhibit differentiation	^82^
Cia-cGAS	Homo sapiens	HSC	Bind to cGAS	TypeⅠIFNs	Maintain the homeostasis in terms of cGAS-mediated HSC exhaustion	^84^
Hsa_circ_105039	Homo sapiens	iPSC	Sponge miR-17	CyclinD2	Promote cardiomyocyte differentiation	^89^
CircRNA Igf1r	Mus	Germline stem cell	Sponge miR-15a-5p,	Inha, Acsl3, Kif21b, Igfbp2	Promote germline stem cell differentiation, sex differentiation, reproductive process, etc.	^90^
CircGFRalpha1	Mus	Germline stem cell	Sponge miR-449	GFRalpha1, GDNF signaling pathway	Promote female germline stem cell self-renewal and express stage-specifically	^91^
CDR1as	*Capra hircus*	SMSC	Sponge miR-7	IGF1R	Promote SMSC differentiation	^92^
CircTMTC1	Gallus gallus	SMSC	Sponge miR-128-3p	Unknown	Inhibit SMSC differentiation	^93^
CircFAM188B	Gallus gallus	SMSC	Encode circFAM188B-103aa	Unknown	Inhibit SMSC differentiation	^94^
CircFNDC3AL	Gallus gallus	SMSC	Sponge miR-204	BCL9	Promote SMSC differentiation	^95^
CircPPP1R13B	Gallus gallus	SMSC	Sponge miR-9-5p	IGF/PI3K/AKT pathway	Promote SMSC differentiation	^96^
Circ-HIPK3	Mus	Myoblast	Sponge miR-124 and miR-379	Unknown	Promote myoblast differentiation	^97^
CircMYBPC1	Bos taurus	Myoblast	Sponge miR-23a	MyHC	Promote myoblast differentiation	^98^
CircZfp609	Mus	Myoblast	Sponge miR-194-5p	BCLAF1	Inhibit myoblast differentiation	^99^
CircTTN	Bos taurus	Myoblast	Sponge miR-432	IGF2/PI3K/AKT Pathway	Promote myoblast differentiation	^37^
CircRILPL1	Bos taurus	Myoblast	Sponge miR-145	IGF1R/PI3K/AKT pathway	Promote myoblast differentiation	^39^
CircSVIL	Gallus gallus	Myoblast	Sponge miR-203	MEF2C and c-JUN	Promote myoblast differentiation	^32^
CircHIPK3	Mus	Myoblast	Sponge miR-7	TCF12	Promote myoblast differentiation	^33^
	Gallus gallus		Sponge miR-30a-3p	MEF2C	Promote myoblast differentiation	^34^
CircITSN2	Gallus gallus	Myoblast	Sponge miR-218-5p	LMO7	Promote myoblast differentiation	^100^
CircSNX29	Bos taurus	Myoblast	Sponge miR-744	Wnt5a/Ca (2+) signaling pathway	Promote myoblast differentiation	^47^
CircFUT10	Bos taurus	Myoblast	Sponge miR-133a	Unknown	Promote myoblast differentiation	^48^
CircMYL1	Bos taurus	Myoblast	Sponge miR-2400	Unknown	Promote myoblast differentiation	^46^
CircEch1	Bos taurus	Myoblast	Unknown	Unknown	Promote myoblast differentiation	^100^
CircHUWE1	Bos taurus	Myoblast	Sponge miR-29b	AKT signaling pathway	Inhibit myoblast differentiation	^36^
CircLMO7	Bos taurus	Myoblast	Sponge miR-378a-3p	Unknown	Inhibit myoblast differentiation	^102^
CircRNA_33287	Homo sapiens	MSMSC	Sponge miR-214-3p	RUNX3	Promote osteogenic differentiation	^103^
CircRNA-1926	*Capra hircus*	SHF stem cell	Sponge miR-14a/b-3p	CDK19	Promote SHF stem cell differentiation	^104^
CircRNA-1967	*Capra hircus*	SHF stem cell	Sponge miR-93-3p	LEF1	Promote SHF stem cell differentiation	^105^
CircSIPA1L1	Homo sapiens	SCAP	Sponge miR-204-5p	ALPL	Promote osteogenic differentiation	^108^
Hsa_circ_0002468	Homo sapiens	SH-SY5Y cell	Sponge miR-561	E2F8	Promote neural differentiation	^111^

ADSC, adipose-derived stem cell; BCLAF1, BCL2-associated transcription factor 1; BMP2, bone morphogenetic protein 2; BMPs, bone morphogenetic proteins; BMSC, bone marrow-derived mesenchymal stem cell; CBFB, core-binding factor subunit beta; CDK19, cyclin dependent kinase 19; c-JUN, c-Jun N-terminal kinase; CTNNB1, catenin beta 1; DPSC, dental pulp stem cell; ESC: embryonic stem cell; FZD4, frizzled class receptor 4; GDF5, growth differentiation factor 5; HSC: hematopoietic stem cell; hucMSC, mesenchymal stem cells derived from human umbilical cord; IFN, interferon; Igfbp2, insulin like growth factor binding protein 2; IGF, insulin like growth factor; IGF1R, insulin like growth factor 1 receptor; IGF2, insulin-like growth factor 2; iPSC, induced pluripotent stem cell; Itga5, integrin alpha 5; KLF4, kruppel like factor 4; LEF1, lymphoid enhancer binding factor 1; MDM2, murine double minute 2; MEF2C, myocyte enhancer factor 2C; MSMSC, maxillary sinus membrane stem cell; MyHC, myosin heavy chain; PDLSC, periodontal ligament stem cell; PI3K, phosphatidylinositol 3-kinase; PTBP1, polypyrimidine tract-binding protein 1; PTEN, phosphatase and tensin homolog; RBPJ, recombination signal-binding protein for immunoglobulin kappa J region; RUNX1, runt-related transcription factor 2; RUNX2, runt-related transcription factor 2; RUNX3, runt-related transcription factor 3; SCAP, stem cell from apical papilla; SHF, secondary hair follicle; SMSC, skeletal muscle satellite cell; STFs, stemness transcription factors; TCF12, transcription factor 12; VEGF, vascular endothelial growth factor; WNT5B: wnt family member 5B; ZEB1, zinc finger e-box binding homeobox 1.

### The effects of circRNAs on stem cell migration

Cell migration is fundamental for development and participates in certain physiological processes, such as the immune response, wound repair and tissue homeostasis.^112^ In stem cells, migration is an inherent ability, similar to self-renewal and differentiation. Stem cell migration is a basic characteristic that is necessary to execute their functions. Enhancement or inhibition of this process can provide insights for the treatment of many diseases.^113^ For example, during wound healing, ADSCs can be recruited promptly to wound sites due to their intense migratory ability. Then, ADSCs begin to differentiate into dermal fibroblasts (DFs), endothelial cells, and keratinocytes.^114^ Several studies have confirmed that circRNAs can alter stem cell migration. In ADSCs, circ_0004303 was upregulated after ADSCs were treated with adenocarcinoma gastric cell (AGS)-derived exosomes. Increased circ_0004303 can sponge miR-148a-3p to increase the expression of activated leukocyte cell adhesion molecule (ALCAM), which is essential for migration and invasion. Therefore, ADSC migration can be enhanced by circ_0004303.^115^ In PDLSCs, circMAP3K11 can accelerate proliferation and migration, but inhibit apoptosis under inflammatory conditions. By sponging miR-511-3p, the expression of TLR4 is elevated; therefore, circMAP3K11 plays a positive role in the migration of PDLSCs.^21^ CDR1as can also contribute to PDLSC migration by maintaining the levels of stemness-related genes. CDR1as sponges miR-7 and increases KLF4 expression to improve PDLSC stemness, including migration and multidifferentiation. Moreover, the RBP hnRNPM can bind to CDR1as and regulate this process.^75^ In hepatocellular carcinoma cancer stem cells (CSCs), circZKSCAN1 can negatively regulate stemness which migration was included. CircZKSCAN1 can bind to an RBP called FMRP and inhibit the expression of CCAR1, which can activate the Wnt signaling pathway. In addition, the RNA-splicing protein Quaking 5 (Qki5) can enhance this process by promoting the production of circZKSCAN1.^116^ Therefore, circRNAs may be therapeutic targets for controlling stem cell migration. However, the relationship between circRNAs and stem cell migration remains unclear.

## CircRNAs regulate the aging and apoptosis in stem cells

Aging and apoptosis are normal life processes and are intricately linked with growth and development. These processes are irreversible, but we can accelerate or alleviate these processes to achieve some therapeutic goals. Therefore, it is necessary to understand the intrinsic mechanisms of aging and apoptosis. Although relevant studies have made significant progress, the roles of circRNAs in aging and apoptosis remain unclear. Here, we summarize previous studies on the effects of circRNAs on these processes.

### The effects of circRNAs on stem cell aging

By using high-throughput RNA sequencing, a study revealed that 4229 circRNAs were involved in age-related senescence in MSCs. Compared with those in the young group, there were 29 differentially expressed circRNAs in the aged group, of which 4 were upregulated and 25 were downregulated. Age-related circRNAs were identified in the circRNA-miRNA-mRNA interaction network. These circRNAs may take part in some important pathways that are associated with aging.^117^ CircRNA FUT10 was elevated in aged skeletal muscle stem cells (SkMSCs). By sponging miR-365a-3p, circRNA FUT10 can increase the expression of homeobox protein Hox-A9 (HOXA9) because miR-365a-3p can also bind to the 3′-UTR of HOXA9 mRNA. HOXA9 expression is correlated with denervated muscle atrophy.^118^ Therefore, circRNA FUT10 can reduce the proliferation and differentiation of SkMSCs and facilitate SkMSC aging.^119^

### The effects of circRNAs on stem cell apoptosis

As demonstrated previously, circRNAs often play positive regulatory roles in stem cell proliferation and differentiation. However, in stem cell apoptosis, the number of circRNAs that have negative regulatory roles is greater than the number of positive circRNAs at present. In PDLSCs, circMAP3K11 inhibits apoptosis by targeting miR-511-3p to increase TLR4 expression in an inflammatory microenvironment.^21^ In BMSCs, circPVT1 can reduce apoptosis by sponging miR-21-5p, which mediates the Smad7/TGFbeta signaling pathway. And circPVT1 overexpression can attenuate steroid-induced osteonecrosis of the femoral head in rats.^29^ In addition, circ_0000020 can also decrease BMSC apoptosis.^53^ The circRNA AFF4 represses apoptosis in MC3T3-E1 cells by sponging miR-7223-5p.^42^ In hucMSCs, CDR1as can also reduce the apoptosis by maintaining the levels of the STFs.^30^ In myoblasts, circRNAs typically sponge miRNAs to regulate apoptosis. For example, circHUWE1 can sponge miR-29b to activate the AKT signaling pathway and reduce bovine myoblast apoptosis.^36^ In addition, circINSR, circRILPL1 and circCPE suppress myoblast apoptosis by sponging miR-34a, miR-145, and miR-138, respectively.^38^, ^39^, ^40^ In addition to the negative regulatory effect of circRNAs on stem cell apoptosis, proliferation or differentiation is usually promoted within the same cell. These factors contribute to the development of stem cells. CircRNAs can also promote stem cell apoptosis. CircRNA_25487 can sponge miR-134-3p to upregulate the expression of P21, thereby facilitating BMSC apoptosis. Excessive circRNA_25487 inhibited bone repair in trauma-induced osteonecrosis of femoral head.^45^ In PDLSCs, circCDK8 overexpression induces autophagy and apoptosis through mTOR signaling.^76^ Hsa-circ_0003420 can target the mRNA of insulin-like growth factor 2 mRNA-binding protein 1 (IGF2BP1) to decrease IGF2BP1 expression. This decrease results in apoptosis in acute myeloid leukemia stem cells. Moreover, hsa-circ_0003420 inhibits the characteristics of leukemia tumor stem cells.^120^ Therefore, targeting circRNAs to alter stem cell apoptosis may be a useful therapeutic strategy.

## CircRNAs regulate the aberrant changes in stem cells

### CircRNAs can alter the malignant behavior of CSCs

In recent years, circRNAs have been have been shown to be important in the formation and progression of cancer, especially hepatocellular carcinoma, gastric carcinoma and colorectal cancer, which reminds us of the importance of circRNAs in cancer diagnosis and therapy.^121^ CSCs, which possess self-renewal ability, are the main reason for cancer recurrence.^122^ Thus, CSCs are a key target for treating cancer. CircRNAs also play roles in CSCs and alter the malignant behavior of CSCs. Zhao et al found that circATP5B can aggravate the proliferation of glioma stem cells (GSCs) by acting as a sponge of miR-185-5p. MiR-185-5p increases the expression of homeobox B5 (HOXB5), which can activate the JAK2/STAT3 signaling pathway to promote GSC proliferation. RBP SRSF1 can promote this process by binding to circATP5B. Interestingly, HOXB5 can transcriptionally regulate SRSF1 expression, forming a feedback loop to regulate this process in GSCs.^123^ Similarly, circCHAF1A can also take part in a feedback loop to facilitate the proliferation and tumorigenesis of TP53wt GSCs. By sponging miR-211-5p, circCHAF1A contributes to the increased expression of homeobox C8 (HOXC8), which can upregulate murine double minute2 (MDM2) expression and inhibit the antitumor effect of p53 to promote the malignant behavior of GSCs. RBP FMR1 can bind to circCHAF1A to maintain its stability and can also be regulated by HOXC8. These factors form a feedback loop among FMR1, circCHAF1A, miR-211-5p, and HOXC8 in GSCs.^124^ Moreover, the U2AF2/circRNA ARF1/miR-342-3p/ISL2 feedback loop was identified in GSCs and can promote glioma angiogenesis and worsen the prognosis.^125^ Therefore, circRNAs may take part in additional feedback loops to regulate GSCs or other CSCs. Surprisingly, circ-E-Cad can promote GSC tumorigenicity by encoding an E-cadherin protein variant that can activate EGFR-STAT3 signaling.^126^ CircRNAs can also negatively regulate CSCs. In hepatocellular carcinoma CSCs (HCC CSCs), circZKSCAN1 can competitively bind to FMRP to inhibit the malignant behavior of HCC CSCs.^116^ CircMEG3 can restrain the growth of human liver CSCs by inhibiting telomerase activity, and this effect is dependent on HULC and Cbf5.^127^

In addition, circRNAs can also alter the CSC-like properties of cancers. These properties incorporate self-renewal, proliferation, migration, and radioresistance. For example, circFAM73A enhances the CSC-like properties of gastric cancer by targeting miR-490-3p.^128^ In glioma cells, circEPHB4 and circPTN can improve stem-like properties and proliferation by sponging miR-637 and miR-145-5p/miR-330-5p, respectively.^129^^,^^130^ Moreover, by sponging miR-145-5p, circPTN can promote the stemness of GSCs. CDR1as can sponge miR-641 to enhance the expression of HOXA9, thereby stimulating stemness and cisplatin chemoresistance in non-small-cell lung cancer (NSCLC).^131^ In contrast, BMSC-derived exosomal circ_0030167 can inhibit the stem-like properties and tumor progression of pancreatic cancer by sponging miR-338-5p and targeting the wif1/Wnt 8/beta-catenin axis.^132^

Therefore, targeting functional circRNAs to repress CSCs or CSC-like properties in cancer is critical for developing therapeutic strategies.

### CircRNAs can affect stem cell injury

Preventing the progression of cell injury is helpful for protecting normal cells. CircRNAs can alter the extent of stem cell injury. Silencing circRNA Cznf292 can attenuate oxygen-glucose deprivation/reoxygenation (OGD/R)-induced injury in rat NSCs by sponging miR-22. Upregulated miR-22 activates the Wnt/β-catenin and PKC/ERK pathways, which can reduce NSC injury induced by OGD/R.^133^ Irradiation is a useful therapeutic method for tumors but can also injure normal cells and even normal stem cells. Circ-016901 contributes to irradiation-induced injury in BMSCs. Circ-016901 can sponge miR-1249-5p to improve HIPK2 expression, which can aggravate injury in BMSCs.^134^ Furthermore, Wang et al showed that the surviving fraction of circRNA_014511 overexpressing BMSCs was distinctly higher than that of the control group after irradiation. Mechanistically, circRNA_014511 can sponge miR-29b-2-5p to decrease the radiosensitivity of BMSCs by altering the cell cycle and apoptosis.^135^ Although there have been few studies on the effect of circRNAs on stem cell radiosensitivity, circRNAs are a new target to protect normal stem cells against radiotherapy or irradiation. We hope that additional mechanisms of stem cell injury involving circRNAs will be identified and have clinical applications.

## Stem cell-derived exosomal circRNAs and their functions

The past several years have confirmed that exosomes can be broadly involved in many biological processes, including intercellular communication, immune responses, pregnancy, neurodegeneration, and cancer progression.^136^ Exosomes are small extracellular vesicles approximately 40–160 nm in diameter that contain many constituents of a cell, such as nucleic acids, lipids, metabolites, and proteins. CircRNAs can also be contained in stem cell-derived exosomes and exert their functions (Table 3).

**Table 3:** Stem cell-derived exosomal circRNAs and their functions.

Exosomal circRNAs	Original cells	Mechanisms	Target genes or pathways	Effects	Ref
Circ-Fryl	ADSC	Sponge miR-490-3p	SIRT3	Attenuate sepsis-induced lung injury	^137^
Mmu_circ_0000623	ADSC	Sponge miR-125	ATG4D	Prevent liver fibrosis	^138^
Mmu_circ_0000250	ADSC	Sponge miR-128-3p	SIRT1	Promote wound healing in diabetic mice	^139^
CircAkap7	ADSC	Sponge miR-155-5p	ATG12, NRF2	Protect against cerebral ischemic injury	^140^
Mmu_circ_0001359	ADSC	Sponge miR-183-5p	FoxO1	Attenuate airway remodeling	^141^
Circ_100395	ADSC	Sponge miR-141-3p	LATS2	Inhibit the Malignant Transformation in NSCLC	^142^
Hsa_circ_0006859	BMSC	Sponge miR-431-5p	ROCK1	Suppress BMSC osteogenesis and promote BMSC adipogenesis	^143^
CircRNA_0001236	BMSC	Sponge miR-3677-3p	Sox9	Enhance chondrogenesis and suppress cartilage degradation	^144^
Circ_0030167	BMSC	Sponge miR-338-5p	wif1/Wnt 8/beta-catenin axis	Inhibit the malignant behaviours pancreatic cancer cells	^132^
Circ-0001273	hucMSC	Unknown	Unknown	Inhibit myocardial cell apoptosis in an ischemic environment and promote MI repair	^145^
CircHIPK3	hucMSC	Sponge miR-421	FOXO3a	Promote the repair of ischemic muscle injury	^19^
CircLPAR1	DPSC	Sponge hsa-miR-31	Unknown	Promote osteogenic differentiation in recipient homotypic DPSCs	^146^

ADSC, adipose-derived stem cell; ATG12, autophagy-related protein 12; ATG4D, autophagy associated protein 4D antibody; BMSC, bone marrow-derived mesenchymal stem cell; DPSC, dental pulp stem cells; hucMSC, mesenchymal stem cells derived from human umbilical cord; LATS2, large tumor suppressor, homolog 2; MI, myocardial infarction; NRF2, nuclear factor erythroid-2 related factor 2; NSCLC, non-small cell lung carcinoma; ROCK1, rho associated coiled-coil containing protein kinase 1; SIRT1, sirtuin 1; SIRT3, sirtuin 3; Sox9, sex-determining region Y-box 9; wif1, wnt inhibitory factor 1.

Researchers have identified several circRNAs that have significant roles in exosomes derived from ADSCs. These circRNAs include circ-Fryl, mmu_circ_0000623, mmu_circ_0000250, circAkap7, mmu_circ_0001359 and circ_100395. These circRNAs also act as sponges of miRNAs. Exosomal circ-Fryl can sponge miR-490-3p to decrease apoptosis in alveolar epithelial cells and the expression of inflammatory factors, thereby alleviating sepsis-induced lung injury.^137^ Exosomal mmu_circ_0000623 can prevent liver fibrosis by sponging miR-125 to promote autophagy activation.^138^ Exosomal mmu_circ_0000250 also activates autophagy in endothelial progenitor cells by sponging miR-128-3p. Autophagy contributes to wound healing in diabetic mice.^139^ Moreover, exosomal circAkap7 can protect against cerebral injury by increasing ATG12-mediated autophagy and decreasing NRF2-mediated oxidative stress by sponging miR-155-5p.^140^ By sponging miR-183-5p, exosomal mmu_circ_0001359 can increase FoxO1 signaling-mediated M2-like macrophage activation and suppress inflammatory cytokine expression to reduce airway remodeling.^141^ In addition, exosomal circ_100395 can suppress malignant transformation in NSCLC by sponging miR-141-3p.^142^

In exosomes derived from BMSCs, hsa_circ_0006859 can suppress BMSC osteogenesis and promote BMSC adipogenesis by enhancing ROCK1 expression by sponging miR-431-5p.^143^ By sponging miR-3677-3p, exosomal circRNA_0001236 facilitates cartilage-specific gene and protein expression, thereby alleviating cartilage degradation and osteoarthritis progression.^144^ As mentioned previously, BMSC-derived exosomal circ_0030167 can also have a suppressive effect on pancreatic cancer cells by sponging miR-338-5p.^132^

In exosomes derived from hucMSCs, circ-0001273 can inhibit myocardial cell apoptosis in an ischemic environment and facilitate myocardial infarction.^145^ CircHIPK3 can promote the repair of ischemic muscle injury by sponging miR-421. Decreasing miR-421 expression can enhance the expression of FOXO3a, which can prevent pyroptosis in skeletal muscle cells and accelerate ischemic muscle repair.^19^

In addition, DPSC-derived exosomal circLPAR1 can promote osteogenic differentiation in recipient homotypic DPSCs by binding to hsa-miR-31. This binding counteracts the adverse impact of hsa-miR-31 on osteogenesis.^146^

## Concluding remarks

In brief, we summarized recent studies about the effects of circRNAs on stem cells, including biological activities, aging and apoptosis, and aberrant changes. We also introduced the biological roles of stem cell-derived exosomal circRNAs. Thanks to the development of bioinformatics, we recognize the value of circRNAs in stem cells. CircRNAs typically act as sponges of miRNAs and have their own expression patterns in stem cells. Therefore, circRNAs can also be useful targets in diagnosis and stem cell therapy. Altering circRNA expression may change the state of stem cells to meet therapeutic requirements. However, apart from sponging miRNAs, other functions of circRNAs, such as being translated into proteins, regulating transcription and interacting with proteins are not dominant in stem cells at present. Thus, despite many prospective studies, the underlying relationships between circRNAs and stem cells are still unclear. We believe that there will be more effects of circRNAs on stem cells discovered and more mechanisms of circRNAs in the regulation of stem cells.

## Conflict of interests

The authors declare no conflict of interests.

## Funding

This work was supported by 10.13039/501100001809The National Natural Science Foundation of China (No. 82173446).
